# Metabolic Changes Following Smoking Cessation in Patients with Type 2 Diabetes Mellitus

**DOI:** 10.3390/biomedicines12081882

**Published:** 2024-08-17

**Authors:** Stamatina Driva, Aliki Korkontzelou, Serena Tonstad, Nikolaos Tentolouris, Eleni Litsiou, Vasiliki Vasileiou, Alice G. Vassiliou, Vassiliki Saltagianni, Paraskevi Katsaounou

**Affiliations:** 1 Smoking Cessation Outpatient Clinic, Respiratory Department, First Intensive Care Unit, Evangelismos General Hospital, 10676 Athens, Greeceelitsiou@yahoo.gr (E.L.);; 2 Diabetes Centre, First Department of Propaedeutic Internal Medicine, Medical School, National and Kapodistrian University of Athens, Laiko General Hospital, 11527 Athens, Greece; 3 Medical School, National and Kapodistrian University of Athens, 11527 Athens, Greece; 4 Department of Preventive Cardiology, Oslo University Hospital, 0424 Oslo, Norway; 5 Endocrinology Department, Diabetes Center, Alexandra Hospital, 11528 Athens, Greece

**Keywords:** smoking cessation, varenicline, weight gain, diabetes, metabolic parameters, appetite-related hormones, physical activity

## Abstract

Background: Smoking cessation is crucial for reducing complications of type 2 diabetes mellitus (T2DM), but associated weight gain can worsen glycemic control, discouraging quitting attempts. Varenicline, a partial agonist of α4β2 nicotinic receptors, aids smoking cessation. This study examines the effects of varenicline on body weight and metabolic parameters in patients with T2DM and prediabetes. Methods: Fifty-three patients were enrolled, of which 32 successfully quit smoking after a three-month course of varenicline and were examined after an additional month with no medication. Measurements taken at baseline, 2.5 months, and 4 months included body weight, blood pressure, resting metabolic rate (RMR), glycated hemoglobin (HbA1c), fasting glucose, blood lipids, C-reactive protein (CRP), appetite-related hormones, and physical activity. Results: Post-treatment, there were no significant changes in body weight, blood pressure, RMR, or glycemic control. Total (CHOL) and low-density lipoprotein (LDL-C) cholesterol decreased significantly at 4 months of the study (from 168 to 156 mg/dL, *p* = 0.013, and from 96 to 83 mg/dL, *p* = 0.013, respectively). Leptin levels increased (from 11 to 13.8 ng/dL, *p* = 0.004), as did glucagon-like peptide-1 (GLP-1) levels (from 39.6 to 45.8 pM, *p* = 0.016) at 4 months of follow-up. The percentage of participants who reported moderate-intensity activity increased from 28% to 56%, while those reporting high-intensity activity increased from 19% to 22%, respectively (*p* = 0.039). Conclusions: Our study showed that smoking cessation with varenicline in smokers with T2DM and prediabetes led to significant improvements in lipid profile, significant increase in plasma leptin and GLP-1 levels, and increased physical activity, without significant weight gain. Thus, smoking cessation without weight gain or deteriorated glycemic control is feasible for these smokers, with added benefits to lipid profiles, GLP-1 regulation, and physical activity.

## 1. Introduction

Cigarette smoking has been shown to aggravate insulin resistance and impair glycemic control in people with type 2 diabetes mellitus (T2DM) [1]. In addition, smokers with T2DM have higher glycated hemoglobin (HbA1c) levels, are more likely to experience severe hypoglycemia, and have difficulties with insulin dose adjustment and diabetes control [2,3,4]. In combination with the harmful effects of glucose dysregulation, smoking is associated with an increased risk of microvascular and macrovascular complications, cardiovascular events, and death in these patients [5,6,7]. Therefore, smoking cessation is highly recommended as a key factor in reducing morbidity and mortality for all patients with diabetes [8].

Post-smoking-cessation weight gain (PSCWG), which occurs mainly due to the cessation of nicotine’s effects on the central nervous system, despite not negating the cardiovascular benefits, may temporarily exacerbate diabetes by aggravating glycemic control [9,10,11], dissuading smokers with T2DM from quitting and increasing recurrence rates [2]. Among smokers with diabetes mellitus (DM), most of the existing literature on the amount of PSCWG is extrapolated from studies in the general population. In two large prospective cohort studies among U.S. men and women with T2DM, the median PSCWG within 6 years was 3.2 kg [12]. Tonstad et al. concluded that mean body weight change from baseline to week 12 in quitters with DM (1.7 kg) was similar to that of those without DM (2.1 kg) in data extracted from 15 double-blind, randomized, placebo-controlled studies [13].

There are numerous studies concerning smoking cessation approaches and their effect on PSCWG in the general population, but few studies have focused on people with diabetes. A systematic review and meta-analysis that evaluated the effects of more intensive compared with less intensive interventions on smoking cessation in people with DM found only a few trials, with no evidence of efficacy [4]. Nicotine increases energy expenditure and resting metabolic rate and suppresses appetite. The appetite-suppressing effects of nicotine are attributed to its complex interactions with various peptides and hormones that regulate food intake and body weight [14,15]. These include adipokines such as leptin and adiponectin, as well as other metabolic hormones like ghrelin, resistin, insulin, glucagon-like peptide 1 (GLP-1), and peptide YY (PYY). These hormones are also crucial in the pathophysiology of T2DM [16]. Therefore, understanding how their levels change after smoking cessation in individuals with T2DM would be of significant interest. Varenicline is a partial agonist of α4β2 nicotinic receptors, resulting in similar effects to nicotine, and is used as a pharmaceutical intervention for smoking cessation. It has been successfully and safely tested for smoking cessation in patients with DM [13,17]. However, there arelittle data regarding its effect on body weight, appetite, insulin sensitivity, and basal metabolism in this population group.

The aim of the present study was to investigate the impact of smoking cessation, aided by varenicline, on body weight, metabolic parameters, lipid and glycemic profiles, and regulatory peptides in individuals with T2DM or prediabetes. To our knowledge, studies in the international literature do not examine all the parameters we study prospectively in this research in combination (somatometric characteristics, laboratory parameters, resting metabolic rate, appetite-related hormones). In addition, existing literature primarily focuses on smoking cessation in healthy individuals or other populations, making this study particularly valuable for understanding the effects of smoking cessation in a sensitive population such as individuals with T2DM, who require ongoing encouragement from healthcare professionals to quit smoking.

## 2. Materials and Methods

### 2.1. Subjects

The study recruited individuals of both sexes, aged 18–70 years, diagnosed with T2DM or prediabetes, who had smoked more than 10 cigarettes in the past 10 days and expressed a willingness to quit smoking. The study’s exclusion criteria included participation in concurrent smoking cessation programs or use of alternative methods (e.g., nicotine replacement therapies), major psychiatric disorders, alcohol consumption exceeding 14 alcohol units per week (1 unit equal to 8 g of alcohol), pregnancy, breastfeeding, illegal substance use, severe kidney failure, thyroid dysfunction, and the initiation or alteration of medications affecting appetite during the study. T2DM was determined based on medication use, while prediabetes was identified by criteria including impaired fasting glucose (IFG), impaired glucose tolerance (IGT), and/or HbA1c levels ranging from 5.7% to 6.4% (39 to 47 mmol/mol), in the absence of antidiabetic medications [18]. All participants received varenicline at no cost as part of the standard protocol for a duration of 3 months, along with counseling sessions. Counseling sessions were provided weekly for the first month and then monthly until the end of the program, as part of an intensive multidisciplinary smoking cessation program. This program was overseen by a team including psychologists, pulmonologists, and diabetologists. The dosage of varenicline followed the medication’s official instructions, gradually increasing to 1 mg twice daily over the course of treatment: starting with 0.5 mg once daily for the initial 3 days, followed by 0.5 mg twice daily for the subsequent 4 days, and finally maintaining a dosage of 1 mg twice daily until treatment completion [19].

The participants were recruited from the Outpatient Internal Medicine—Diabetes and Endocrinology Clinics of the “Laiko”, “Evangelismos”, and “Pammakaristos” Hospitals in Athens, Greece. A total of 53 patients with T2DM and prediabetes were initially enrolled in the study, with 32 successfully quitting smoking and completing follow-up. All participants had signed an informed consent form before participating in the study. Twenty-one patients discontinued participation due to failure to quit smoking with varenicline, significant reduction in cigarette consumption without complete cessation, or persistent severe nausea despite medication adjustments. Participants who completed the study did not alter their pre-existing antidiabetic medication regimen throughout the duration of the study. Among them, three had prediabetes and managed it solely through dietary interventions, while the remaining 29 had T2DM. Among those with T2DM, 100% were on metformin, with 7% on sulfonylureas, 35% on dipeptidyl peptidase-4 (DPP-4) inhibitors, 7% on glucagon-like peptide-1 receptor (GLP-1R) agonists, 14% on sodium-glucose co-transporter-2 (SGLT2) inhibitors, and 14% on insulin therapy solely or in combination with oral antidiabetic agents.

### 2.2. Description of Study Procedures

**Figure 1:**
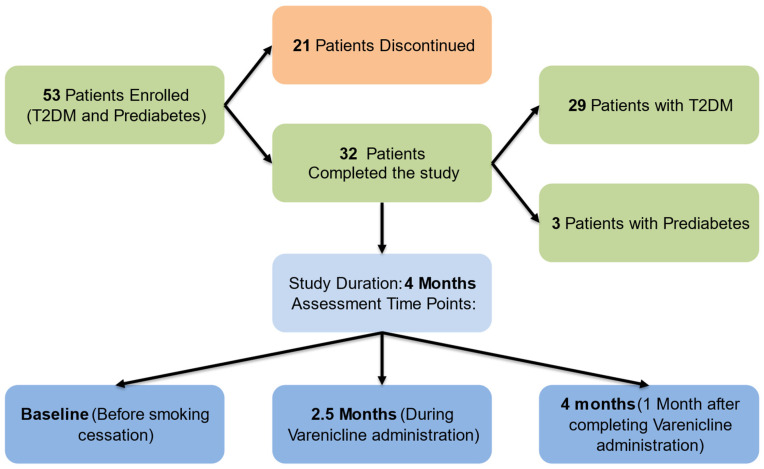
Study Flow Chart.

The study design involved a 4-month investigation into the effects of varenicline on various parameters in smokers with T2DM and prediabetes. During the 4 months of the study, participants underwent assessments at three time points: before smoking cessation (baseline); at 2.5 months of drug administration, while the participants had stopped smoking and continued to take the medication; and at 4 months after the beginning of the study and specifically 1 month after completing varenicline administration. The study flow chart is presented in Figure 1. This comprehensive approach allowed for the evaluation of varenicline’s impact over time on the measured parameters. By including a follow-up period post medication cessation, we were able to observe the sustainability of any observed effects.

The assessments included measurement of somatometric characteristics such as body weight, height, waist (WC), and hip circumference (HC), from which the body mass index (BMI) and waist-to-hip ratio (WHR) were calculated as the quotient of the weight in kg divided by the square of the height in meters and the ratio of waist circumference to hip circumference, respectively. Blood pressure was measured three times at a seated position using the left arm, with the average recorded. The resting metabolic rate (RMR) was determined using a “Vmax Spectra 22” machine (SensorMedics Corporation, Anaheim, CA, USA), and exhaled carbon monoxide (CO) levels were measured with a “Micro + Smokerlyzer” meter (Bedfont Scientific Ltd., Maidstone, Kent, UK). Participants completed the International Physical Activity Questionnaire (IPAQ 2002) [20] and the Fagerström Test for Nicotine Dependence (FTND) [21] to assess physical activity and nicotine dependence, respectively. The IPAQ score yields two types of output. The results were presented as a continuous variable (metabolic equivalent of task—MET minutes per week) or as categories (low, moderate, or high activity levels). MET minutes represent the amount of energy expended carrying out physical activity.

Blood samples were also collected early in the morning after 8–10 h fasting from each participant at three separate times during the study. These samples were used to measure various parameters, including HbA1c, glucose, lipid profile (total cholesterol, high-density lipoprotein cholesterol, low-density lipoprotein cholesterol, triglycerides), C-reactive protein (CRP), urea, creatinine, and estimated glomerular filtration rate (eGFR), using the CKD-EPI 2021 method [22]. Additionally, 4 mL of additional blood was collected in two tubes containing EDTA. In one tube, DPP-4 inhibitor was added, and after centrifugation, the plasma was stored at −80 °C for later analysis of leptin, adiponectin, resistin, insulin, GLP-1, and PYY. In the other tube, plasma was collected, and after the addition of phenylmethylsulfonyl fluoride (PMSF) and hydrochloric acid (HCL), the mixture was vortexed and centrifuged, and the clear content was collected and stored at −80 °C for subsequent measurement of ghrelin levels. Hormone levels were measured using enzyme-linked immunosorbent assays (ELISA) according to the manufacturers’ instructions (Merck Millipore, Burlington, MA, USA). The detection limits for leptin, GLP-1, ghrelin, adiponectin, resistin, PYY, and insulin were 0.2 ng/mL, 1.5 pM, 50 pg/mL, 0.2 ng/mL, 0.02 ng/mL, 6.5 pg/mL, and 1 μu/mL, respectively. Intra-assay coefficients of variation (CV) were <4.9%, and inter-assay CVs were <8.6% for leptin, <2% and <12% for GLP-1, <1.9% and <7.8% for ghrelin, <7.4% and 8.4% for adiponectin, <7% and <7.7% for resistin, <5.7% and <6.9% for PYY, and <6.9% and <11.4% for insulin. These results indicate that the test method reliably detected hormone levels. The homeostasis model assessment for insulin resistance (HOMA-IR) was calculated as fasting immunoreactive insulin (μu/mL) multiplied by fasting plasma glucose (mg/dL) divided by 405.

Thyroid hormones (thyroid-stimulating hormone TSH, triiodothyronine T3, free thyroxine fT4) were assessed only during the screening visit, as dysregulated thyroid function was an exclusion criterion from the study. If the initial visit and blood tests revealed no exclusion criteria, such as severe renal impairment (GFR < 30 mL/min/1.73 m^2^) or thyroid dysfunction, participants commenced varenicline treatment the following day.

### 2.3. Statistical Analysis

Quantitative variables with a normal distribution were expressed as mean ± standard deviation, while those without normal distribution were presented as median with interquartile range (25%, 75%). Qualitative variables were represented as percentages (n %). Normality of variables was assessed by calculating differences between variables at different time points and examining histograms for normal distribution. Additionally, the Shapiro–Wilk test was employed. Parametric tests were applied for normally distributed variables, while non-parametric tests were used for non-normally distributed variables.

The changes in the variables were analyzed in pairs by comparisons between the three time points of the measurements. Paired samples *t*-tests were performed for normally distributed quantitative variables, whereas the Wilcoxon matched-pair signed-rank test was employed for non-normally distributed quantitative variables. The McNemar–Bowker test (crosstabulation) was used to analyze the results of the qualitative variable IPAQ. *p* values less than 0.05 were defined as the level of statistical significance.

To assess correlations between changes in plasma peptide levels and changes in weight-related parameters from the inclusion visit to the end of follow-up, we applied Spearman correlation analysis, since most factors deviated from a normal distribution. Adjustments for multiple comparisons were performed using the Bonferroni correction method. The statistical analysis was conducted using IBM SPSS Statistics 26.

## 3. Results

### 3.1. Anthropometric and Biochemical Parameters over Time

The baseline demographic characteristics and laboratory data of the 32 participants who completed the study are presented in Table 1. The mean age was 60 ± 7.6 years, and the median duration of DM was 5 (2; 10) years. Of the 32 diabetic smokers, 19 were men, and 13 were women, and most of the participants (72%) were taking medication for arterial hypertension. The median number of cigarettes smoked per day was 28 (20; 40), and the median FTND score was 9 (8; 10), indicating a high level of nicotine dependence among the subjects. The average BMI was 30.1 ± 6 kg/m^2^, RMR averaged 1441 ± 308 kcal/day, and the median HbA1c level was 6.0% (with a range of 5.7% to 6.8%). Participants demonstrated favorable kidney and thyroid function, as evidenced by the observed GFR and TSH values.

Table 2 presents the values in somatometric and laboratory characteristics of the 32 individuals who successfully completed the study. The comparisons involve data from the baseline visit with the 2.5-month visit, the baseline visit with the 4-month visit, and the 2.5-month visit with the 4-month visit.

From our results, we observe that after completion of varenicline treatment, body weight and BMI did not show statistically significant changes. This lack of significant change also extends to WC, WHR, blood pressure, RMR, and glycemic control. Specifically, when comparing median glucose and HbA1c values between the baseline visit (0) and the 2.5-month treatment period (*p* = 0.416 and 0.470, respectively), as well as between the baseline visit (0) and the 4-month follow-up (*p* = 0.137 and 0.382, respectively), no significant alterations were observed.

In contrast, CRP decreased significantly with smoking cessation at 2.5 months of the intervention (*p* = 0.031), a change that did not persist at 4 months after the beginning of the study (*p* = 0.546). Of particular interest is the improvement in the lipid profile. Total cholesterol (CHOL) and low-density lipoprotein cholesterol (LDL-C) decreased significantly at 2.5 months (from 168 to 147 mg/dL, *p* = 0.001, and from 96 to 71 mg/dL, *p* < 0.001, for CHOL and LDL-C, respectively), as well as at the 4-month follow-up after smoking cessation (from 168 to 156 mg/dL, *p* = 0.013, and from 96 to 83 mg/dL, *p* = 0.013, for CHOL and LDL-C, respectively). In addition, high-density lipoprotein cholesterol (HDL-C) increased significantly at 2.5 months of treatment (from 44 to 45 mg/dL, *p* = 0.042), and a decline in the value of triglycerides (TG) was observed during and after smoking cessation, but this reduction was not significant (from 143 to 124 mg/dL, *p* = 0.379, at 2.5 months of treatment and from 143 to 125 mg/dL, *p* = 0.235, at 4 months of follow-up).

**Figure 2:**
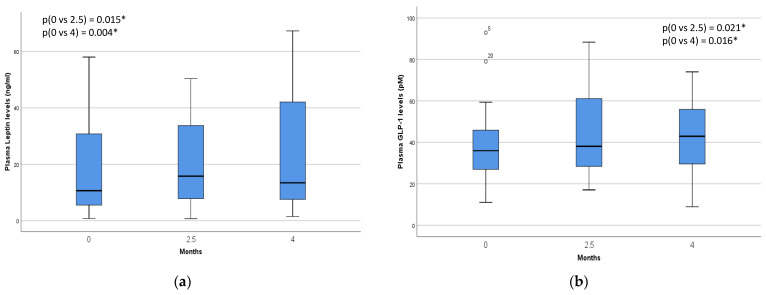
The box plots show the median and quartiles, and the whisker caps of the box plots show the mean 5th and 95th percentile values. A Wilcoxon matched-pair signed-rank test yielded the results. (**a**) The plasma levels of leptin at baseline (0), 2.5 months, and 4 months were 11 (5.4; 33.7), 17 (7.6; 39.4), and 13.8 (7.5; 44) ng/L, respectively. (**b**) Fasting GLP-1 levels at baseline (0), 2.5 months, and 4 months were 39.6 (27.7; 58.5), 41.8 (29.3; 70.7), and 45.8 (31.1; 69.1) pM, respectively. * *p* values with Wilcoxon matched-pair signed-rank test. Data are presented as median (interquartile range).

We next measured the levels of peptides that are associated with appetite and body composition. Leptin levels demonstrated a significant increase throughout the smoking cessation program (*p* = 0.015), a change that persisted beyond the conclusion of varenicline treatment (*p* = 0.004). This trend was similarly observed in GLP-1 levels, with values increasing from 39.6 to 41.8 pM at 2.5 months of treatment (*p* = 0.021) and from 39.6 to 45.8 pM at 4 months of follow-up (*p* = 0.016). Figure 2 shows the plasma leptin and GLP-1 levels at each point in time for the 32 successful participants. In contrast, the levels of the other hormones and the HOMA-IR did not exhibit significant changes during the study.

Finally, the level of exhaled CO decreased significantly both during and after the smoking cessation therapy (*p* < 0.001). This not only signifies positive pulmonary outcomes for the patient but also serves as compelling evidence of smoking cessation.

### 3.2. Increase in Self-Reported Level of Physical Activity Intensity, According to IPAQ 2002

**Figure 3:**
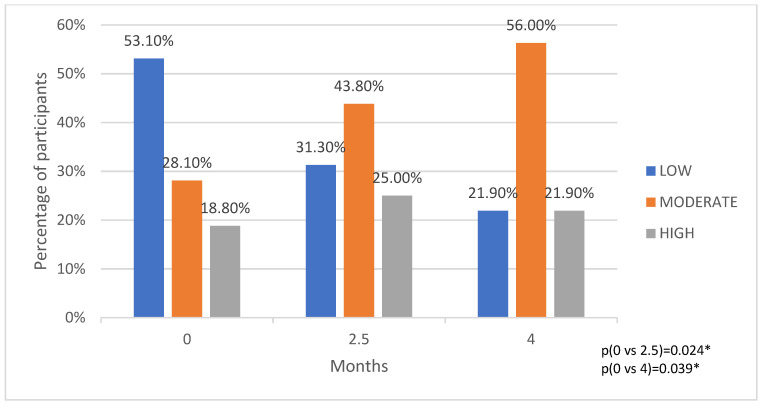
International Physical Activity Questionnaire (IPAQ 2002) and comparative data for self-reported level of physical activity intensity (low, moderate, high). * *p* values with McNemar–Bowker test (crosstabulation).

From the analysis of the responses of the 32 participants to the IPAQ, which was completed at each of the three visits, there appeared to be an increase in physical activity intensity level throughout the program, with a decrease in the percentage of participants who reported low-intensity activity and an increase in the percentage of participants who reported moderate and high-intensity activity. The percentage of participants who reported moderate-intensity activity increased from 28.1% at the start of the study to 43.8% at 2.5 months of varenicline administration and then to 56% at the last visit (Figure 3). Comparing the median values of the continuous quantitative variable MET-minutes per week (MET-min/week) during the study, we also observed a statistically significant increase in the level of physical activity (Table 3).

### 3.3. Correlations between Appetite-Related Hormones and Weight-Related Parameters over Time

Table 4 presents significant correlations between changes in plasma peptide levels and changes in weight-related parameters from the inclusion visit to the end of the follow-up. As anticipated, there was a correlation between the rise in leptin levels and the increases in body weight, BMI, and WC. The increase in GLP-1 levels presents a negative correlation with CHOL and LDL-C levels over time. In other words, the higher the GLP-1 levels, the more likely there was a decrease in total and LDL-C levels. The change in plasma ghrelin levels was found to be negatively correlated with body weight, BMI, CHOL, LDL-C, and TG changes over time, as expected. Adiponectin levels showed a positive correlation with the increase in HDL-C, while PYY levels exhibited a negative correlation with changes over time in CHOL and LDL-C. No statistically significant correlation was identified between resistin and HOMA-IR changes and alterations in weight-related parameters from the study’s inclusion to its final 4-month visit. Additionally, the correlation between change in MET-min/week and changes in peptide levels over time showed no significant relationship, indicating that improvements in physical activity did not affect the results. We also examined correlations between changes in hormone levels with each other, as well as the correlation between the increase in MET-min/week and changes in other weight-related parameters such as BMI, RMR, WC, and lipid parameters but found no significant correlations. These findings are not presented in the table.

## 4. Discussion 

The purpose of the present study was to examine the effect of smoking cessation with the use of varenicline on various metabolic parameters in smokers with T2DM and prediabetes. The main findings of this study were that smoking cessation with the use of varenicline resulted in significant amelioration in the lipid profile of the participants, significant increases in plasma leptin and GLP-1 levels, and a significant increase in the self-reported level of physical activity. In addition, there was no significant PSCWG. Notably, these changes persisted even one month after the end of varenicline treatment.

Cigarette smoking is widely recognized as negatively impacting lipid profiles, leading to increased triglycerides and cholesterol lipoproteins [23,24,25]. This effect stems from smoking’s interference with the cytochrome enzyme system involved in lipid and cholesterol metabolism and transport [26]. Previous studies consistently show that current smokers tend to have lower HDL cholesterol levels compared to ex-smokers and non-smokers [25,27,28] and higher triglyceride concentrations compared to non-smokers, regardless of BMI [29]. Regarding patients with T2DM, the LDL-C: HDL-C ratio and the log-transformed triglycerides: HDL-C ratio tend to be higher with an increase in the amount of smoking [30]. Smoking cessation improves lipid and metabolic profiles, as demonstrated by decreases in total cholesterol and increases in HDL cholesterol observed just one month after quitting, according to recent research [31]. Studies on smoking cessation with varenicline have revealed significant reductions in LDL-C levels [32], along with decreases in oxidized high-density lipoprotein (oxHDL) levels—a potential marker for cardiovascular diseases—coupled with significant increases in HDL-C and improvements in serum apolipoprotein apoA-I levels in the short term [33,34]. Additionally, despite weight gain, cessation improves HDL cholesterol, total HDL, and large HDL particle concentrations, as well as enhancing HDL-C functionality [35,36,37]. Moreover, quitting smoking is associated with favorable changes in triglyceride concentrations and diastolic blood pressure, as evidenced by a randomized clinical trial involving overweight and obese smokers receiving dietary advice and varenicline [38]. On the other hand, several studies have shown no significant alterations in lipid profiles following smoking cessation [25,32,35,37,39]. These conflicting findings may be attributed to variations in body weight, diet, and duration of cessation.

In our study, the improvement in the lipid profile is demonstrated by statistically significant reductions in total and LDL-C levels throughout the program, as well as increases in HDL-C levels at 2.5 months of treatment, although this change did not persist at the 4-month follow-up. Following the comparison of the parameters’ values between the 2.5-month and 4-month time points, during which no significant change was observed, we concluded that the positive changes in the values of CHOL and LDL-C were maintained at the end of the smoking cessation program. To our knowledge, although the above studies showed associations between smoking or smoking cessation and lipid panel changes, our study uniquely examined the impact of smoking cessation with varenicline on the lipid profiles of individuals with T2DM.

As far as the other hormones are concerned, this is the first study that comprehensively examined them simultaneously in a smoking cessation program in people with T2DM. In our prior research, we conducted an extensive review of global literature exploring the influence of smoking cessation on peptides involved in regulating food intake and body weight [15]. In summary, our findings indicated that smoking cessation is associated with elevated leptin levels and increase in serum adiponectin levels in individuals with less abdominal obesity. However, conflicting data exist regarding the effects of smoking cessation on plasma levels of ghrelin, while no significant impact of cessation was noted on PYY, GLP-1, and CCK levels [15].

In the present study, we found a substantial rise in plasma leptin levels throughout the study duration, and this increase correlated positively with changes in BMI, body weight, and WC. Leptin, an adipose-tissue-derived hormone, plays a crucial role in regulating satiety and thermogenesis [40]. It acts by signaling to the brain the saturation of fat cells, thereby reducing hunger and helping to maintain a healthy weight [41]. Leptin secretion is directly proportional to body weight and fat mass [42], making it positively correlate with waist circumference [43,44]. Most of the studies note a significant increase in serum leptin levels after smoking cessation [45,46,47,48]. This increase is positively correlated with increases in body weight, BMI, and body fat mass [40,45,47,49,50,51,52]. Conversely, other publications have failed to find an increase in leptin levels following smoking cessation, despite the observed weight gain [53]. Such disparities may arise due to the intricate interplay between smoking and various factors such as diet, exercise, hormones, and inflammatory responses, which can impact leptin regulation [54]. A recent meta-analysis involving 40 studies revealed a significant difference in serum leptin levels between smoking and non-smoking groups, with lower leptin levels observed in the smoking group overall. However, subgroup analyses based on study design and population health status yielded varied results, indicating the complex nature of the relationship between smoking and leptin levels [55].

GLP-1 is an incretin hormone secreted postprandially by intestinal enteroendocrine L-cells and neurons in the nucleus tractus solitarius of the caudal brainstem [56]. It acts by enhancing insulin secretion and suppressing glucagon release, thereby regulating blood sugar levels in a glucose-dependent manner [57]. Additionally, GLP-1 inhibits appetite, promotes satiety, delays gastric emptying, improves insulin sensitivity, stimulates beta-cell regeneration, and prevents beta cell apoptosis, making it a promising target for diabetes and obesity treatment [58]. Most studies focus on investigating GLP-1R agonists as a potential treatment for smoking cessation, suggesting their effectiveness in controlling PSCWG and regulating glucose metabolism [59,60,61]. However, there is limited evidence regarding the direct impact of nicotine and smoking cessation on GLP-1 levels in humans [62]. In a study conducted by Stadler et al., fasting plasma concentrations of GLP-1, ghrelin, visfatin, and PYY were not significantly changed three months after smoking cessation compared with the baseline [53]. Similarly, levels of gastric inhibitory polypeptide (GIP), GLP-1, amylin, insulin, PYY, and pancreatic polypeptide (PP) measured after a meal challenge were unaffected after three months of abstinence, as reported by Pankova et al. However, it is possible that changes in incretin levels occurred earlier following smoking cessation [45]. More recently, Yannakoulia et al. attempted to elucidate the acute decrease in energy intake associated with acute smoking by investigating hormonal factors. However, they found no significant differences in the changes of blood concentrations of CCK, GLP-1, ghrelin, and obestatin over time between smoking and control conditions [63].

In our study, we observed a significant increase in GLP-1 levels following smoking cessation, suggesting a potentially favorable effect on GLP-1 regulation. This increase was negatively correlated with CHOL and LDL-C levels over time. This relationship is supported by the known metabolic benefits of GLP-1, which include lipid metabolism improvement and cardiovascular protection. Recent work has shown that GLP-1 analogs can reduce CHOL and LDL-C levels, highlighting the potential for GLP-1 to positively influence lipid profiles [64]. In individuals with T2DM, fasting and postprandial GLP-1 levels are typically reduced compared to those with normal glucose tolerance. An increase in fasting GLP-1 levels is generally considered beneficial for individuals with diabetes, as it can help improve glycemic control and metabolic health [57,65]. However, individual responses to GLP-1 modulation may vary, and further research is needed to fully understand its therapeutic potential in diabetes management. While physical activity is recommended for managing T2DM, its impact on GLP-1 concentrations varies [66]. Some studies suggest that high-intensity exercise may be required to increase GLP-1 concentration [66], but our study did not find a correlation between physical activity intensity level and GLP-1 levels. Our findings suggest a potential link between smoking cessation and increased GLP-1 levels, highlighting the need for further investigation into the role of GLP-1 in diabetes management and the effects of lifestyle interventions, such as physical activity, on GLP-1 regulation.

The levels of other hormones and the HOMA-IR did not show statistically significant changes during and after the smoking cessation program, and the same was observed for body weight, BMI, WC, WHR, blood pressure, RMR, and glycemic control. Many studies in the global literature report significant weight gain following smoking cessation [15,67,68], and in a study conducted by Komiyama et al., triglycerides and FTND score were found to be the factors determining the post-cessation BMI increase, with the FTND score being the strongest one [69]. In our study, the change in BMI, which was not significant, was not associated with any somatometric or biochemical variable before cessation. There are findings in alignment with our investigation, where patients with T2DM or obesity receiving varenicline for smoking cessation had no statistically significant PSCWG [38,70,71]. Weight gain following smoking cessation poses a significant concern among individuals with T2DM due to its potential impact on diabetes control with impaired fasting glucose and an increase in HbA1c levels within the first year after quitting [10,72,73]. Stadler et al. [53] reported increased body weight, fat mass, fasting insulin levels, and a deterioration in fasting insulin sensitivity three months after smoking cessation. However, fasting insulin, glucose, HbA1c, and HOMA-IR showed no significant increase throughout our smoking cessation program, which suggests that varenicline is an effective and well-tolerated aid for smoking cessation in people with DM.

The correlations observed between changes in plasma peptide levels and weight-related parameters throughout the study period are consistent with findings from existing literature. Specifically, the negative correlation between changes in plasma ghrelin levels and body weight, BMI, CHOL, LDL-C, and TG changes over time supports the established role of ghrelin in regulating energy balance and metabolism. Ghrelin is known to stimulate appetite and increase food intake, which can impact body weight and fat accumulation [74]. Additionally, our study found a positive correlation between adiponectin levels and increases in HDL-C. This finding aligns with previous research indicating that adiponectin, an adipokine with anti-inflammatory and insulin-sensitizing properties, positively affects lipid profiles and cardiovascular health [75]. Finally, the observed negative correlation between PYY levels and changes in CHOL and LDL-C over time suggests that PYY plays a beneficial role in lipid metabolism. As a gut-derived hormone, PYY is known to reduce appetite and food intake, which contributes to lower body weight and improved metabolic outcomes. Previous studies have demonstrated that PYY can positively influence lipid profiles by decreasing levels of atherogenic lipoproteins such as LDL-C [76].

Regarding physical activity, it is widely known to play a crucial role in promoting health and reducing the risk of non-communicable diseases such as cardiovascular disease, certain cancers, and T2DM [77]. The American College of Sports Medicine and the American Diabetes Association emphasize the importance of exercise for individuals with T2DM, highlighting its role in weight management and weight gain prevention in these patients, who are particularly susceptible to PSCWG [78]. Researchers have investigated whether exercise and increase in physical activity levels can help ameliorate weight gain associated with smoking cessation [79,80,81]. In our study, we noticed an increase in self-reported level of physical activity intensity, according to the IPAQ 2002. The majority of studies in the international literature primarily discuss physical activity as an aid for smoking cessation [82,83,84] rather than its role as an outcome in smoking cessation programs aimed at improving overall well-being and reducing PSCWG. It has been reported that smokers who have low levels of physical activity at baseline are at an increased risk for PSCWG and that it is important to integrate physical activity components into smoking cessation programs [85]. Quitters who increase or maintain a moderate or high intensity level of physical activity experience significantly less weight gain compared to those who decrease their activity levels [86,87,88]. On the other hand, during a 5-year follow-up involving 281 young Australian smokers, quitters tended to adopt healthier dietary and physical activity behaviors compared to continuing smokers, suggesting that these changes did not explain the observed PSCWG [89]. A meta-analysis assessing various interventions, including pharmacological treatments and non-pharmacological programs like weight management education, low-calorie diet, or exercise for preventing PCWG, showed no significant reduction in weight with exercise interventions at the end of treatment. However, a significant weight reduction was observed at 12 months post intervention [80]. Subsequent updates to the review reaffirmed these findings, indicating low certainty regarding the effectiveness of exercise interventions in achieving minimal or no weight reduction compared to standard care at the end of the treatment, though weight reduction was noticeable after 12 months [90]. Thus far, the benefits of physical activity in ameliorating PSCWG are inconclusive.

Our study has several limitations primarily stemming from the small sample size, exacerbated by the impact of the COVID-19 pandemic on participant attendance in the smoking cessation program and finally the withdrawal of varenicline that led to the abrupt termination of the study. With a larger sample, it may be possible that more significant changes in the variables, particularly regarding appetite-regulating peptide levels and their correlations with other variables, could have been detected, shedding light on potential mechanisms underlying PSCWG. Additionally, changes in dietary habits could have influenced hormone levels and other biochemical parameters. Furthermore, no dynamic meal challenges were performed to examine for changes in appetite regulatory hormones during smoking cessation. Despite these limitations, this study lays a foundation for further clinical investigations into the effects of smoking cessation programs on various metabolic parameters in patients with DM, offering insights into a comprehensive approach to their management.

## 5. Conclusions

PSCWG is a major barrier to smoking cessation and often leads to relapse, especially among smokers with T2DM, as it may lead to the deterioration of glycemic control and temporarily exacerbate diabetes control. We believe that our results provide important information, since we showed that smoking cessation without weight gain is feasible for diabetic smokers, with additional benefits for their lipid profile and increased physical activity. Positive changes in CHOL and LDL-C were maintained at the end of the program, enhancing the long-term benefits of smoking cessation.

With regard to practical and clinical perspectives, our findings confirm that smoking cessation using varenicline is a viable option for individuals with T2DM without significant adverse effects on glycemic control, as evidenced by stable fasting insulin, glucose, HbA1c, and HOMA-IR levels. This finding supports the recommendation that healthcare providers encourage diabetic smokers to quit, reassuring them about the manageable risks of PSCWG. The observed improvements in lipid profiles and physical activity levels also highlight the potential for broader health benefits and support the need for an integrated approach that combines smoking cessation programs with dietary counseling and physical activity promotion. Furthermore, our study evaluated a wide range of appetite-related hormones, revealing a substantial increase in plasma leptin levels and a significant rise in GLP-1 levels. These changes indicate a potential positive effect on GLP-1 regulation, which could have implications for managing appetite and metabolic health during smoking cessation. One example could be the use of GLP-1R agonists to prevent weight gain in individuals with T2DM who are trying to quit smoking.

Future research directions should prioritize randomized controlled trials with larger sample sizes and longer follow-up periods to validate the findings and assess the long-term effects of smoking cessation on metabolic parameters in patients with T2DM. Further studies should explore the mechanisms behind the changes in appetite-regulating hormones following smoking cessation. Understanding these mechanisms could reveal potential therapeutic targets to mitigate weight gain and improve metabolic outcomes. Additionally, dynamic meal challenges and dietary interventions during cessation could offer deeper insights into managing weight gain and optimizing metabolic health. Comparative studies across different populations may also provide a richer understanding of the unique challenges faced by diabetic smokers. These insights lay a foundation for a more comprehensive and supportive approach to smoking cessation in patients with T2DM, addressing both the challenges and benefits associated with quitting smoking. Furthermore, while our study focuses on the direct effects of smoking cessation in active smokers, it is important to acknowledge the potential impact of passive smoking on metabolic health. Future research could explore the effects of passive smoke exposure on similar metabolic parameters in individuals with T2DM to inform public health recommendations. Given that most studies focus on healthy individuals or other populations, our findings are particularly relevant for the vulnerable population of individuals with T2DM, where weight gain is strongly related to their disease. Smokers with T2DM should be consistently encouraged to quit smoking by healthcare professionals and informed that post-cessation weight gain may be managed.

## Figures and Tables

**Table 1:** Baseline demographic and laboratory characteristics of participants (n = 32, T2DM = 29, prediabetes = 3).

Variables	Baseline Values
Age (Years)	60 ± 7.6
Sex (Men/Women) n (%)	19/13 (59%)
Duration of DM (years)	5 (2; 10)
Arterial Hypertension (Yes/No) n (%)	23/9 (72%)
Cigarettes/Day	28 (20; 40)
Pack/Years	50 (40; 68)
FTND score	9 (8; 10)
Weight (kg)	86.8 ± 17
BMI (kg/m^2^)	30.1 ± 6
WHR (Ratio)	0.98 ± 0.1
SBP (mmHg)	123 ± 14
DBP (mmHg)	76 ± 9.3
HbA1c (%)	6.0 (5.7; 6.8)
GFR (mL/min)	87.7 ± 19.7
CRP (mg/dL)	0.2 (0.1; 0.7)
T3 (ng/dL)	112 (96; 145)
FT4 (ng/dL)	1.3 (1.2; 1.4)
TSH (μU/mL)	1.3 (1.1; 2.1)
RMR (kcal/day)	1441 ± 308
CO (ppm)	12 (8; 17)

DM: diabetes mellitus; FTND: Fagerström Test for Nicotine Dependence; BMI: body mass index; WHR: waist-to-hip ratio; SBP: systolic blood pressure; DBP: diastolic blood pressure; HbA1c: glycated hemoglobin; GFR: glomerular filtration rate; CRP: C-reactive protein; T3: triiodothyronine; FT4: free thyroxine; TSH: thyroid-stimulating hormone; RMR: resting metabolic rate; CO: carbon monoxide. Data are presented as n (%), mean ± standard deviation, or median (interquartile range).

**Table 2:** Follow-up and comparative data of the 32 participants over the study period.

Variables	Baseline (0)	2.5 Months	4 Months	*p*-Values 0 vs. 2.5	*p*-Values 0 vs. 4	*p*-Values 2.5 vs. 4
Weight (kg)	86.8 ± 17	87.7 ± 17	88.4 ± 17	0.155 *	0.061 *	0.182 *
BMI (kg/m^2^)	30.1 ± 6	30.4 ± 6	30.6 ± 5.5	0.149 *	0.074 *	0.235 *
WC (cm)	104.5 ± 12.6	104.6 ± 11.5	104.1 ± 12	0.916 *	0.690 *	0.389 *
HC (cm)	106.9 ± 9.7	108.1 ±1 0.7	107.7 ± 11.6	0.082 *	0.339 *	0.404 *
WHR (Ratio)	0.98 ± 0.1	0.97 ± 0.1	0.97 ± 0.1	0.292 *	0.259 *	0.965 *
SBP (mmHg)	123 ± 14	123 ± 13	123 ± 14	0.916 *	0.905 *	0.987 *
DBP (mmHg)	76 ± 9.3	74.7 ± 8.8	75.4 ± 7.1	0.431 *	0.595 *	0.549 *
RMR (kcal/day)	1441 ± 308	1453 ± 276	1441 ± 276	0.951 *	0.920 *	0.940 *
Glucose (mg/dL)	107 (94; 129)	115 (99; 136)	116 (92; 145)	0.416 **	0.137 **	0.190 **
HbA1c (%)	6.0 (5.7; 6.8)	6.3 (5.7; 6.9)	6.4 (5.7; 6.8)	0.470 **	0.382 **	0.273 **
CRP (mg/dL)	0.2 (0.1; 0.7)	0.2 (0.1; 0.5)	0.2 (0.1; 0.6)	0.031 **	0.546 **	0.052 **
CHOL (mg/dL)	168 (146; 193)	147 (135; 173)	156 (143; 177)	0.001 **	0.013 **	0.270 **
LDL-C (mg/dL)	96 (73; 120)	71 (57; 97)	83 (68; 95)	0.000 **	0.013 **	0.103 **
HDL-C (mg/dL)	44 (36; 49)	45 (40; 51)	44 (40; 52)	0.042 **	0.190 **	0.588 **
TG (mg/dL)	143 (107; 204)	124 (98; 194)	125 (100; 187)	0.379 **	0.235 **	0.697 **
Leptin (ng/mL)	11 (5.4; 33.7)	17 (7.6; 39.4)	13.8 (7.5; 44)	0.015 **	0.004 **	0.127 **
GLP-1 (pM)	39.6 (27.7; 58.5)	41.8 (29.3; 70.7)	45.8 (31.1; 69.1)	0.021 **	0.016 **	0.804 **
Ghrelin (pg/mL)	522 (403; 730)	503 (368; 608)	513 (378; 656)	0.270 **	0.432 **	0.754 **
Adiponectin (ng/mL)	5617 (3103; 6999)	5359 (2414; 7195)	5057 (2690; 7020)	0.096 **	0.340 **	0.974 **
Resistin (ng/mL)	31.5 (23.3; 41.3)	28.6 (20.7; 37.9)	28.6 (20.5; 37.9)	0.265 **	0.127 **	0.754 **
PYY (pg/mL)	136 (89; 170)	140 (98; 170)	134 (94; 168)	0.231 **	0.360 **	0.905 **
Insulin (μU/mL)	8.2 (5.8; 21.3)	12.6 (7.6; 25.4)	11 (7.2; 20)	0.177 **	0.652 **	0.316 **
HOMA-IR	2.67 (1.82; 5.57)	3.26 (2.01; 7.78)	3.46 (2.03; 6.65)	0.131 **	0.281 **	0.785 **
CO (ppm)	12 (8; 17)	3 (1; 3)	2 (1; 3)	0.000 **	0.000 **	0.437 **

DM: BMI: body mass index; WC: waist circumference; HC: hip circumference; WHR: waist-to-hip ratio; SBP: systolic blood pressure; DBP: diastolic blood pressure; RMR: resting metabolic rate; HbA1c: glycated hemoglobin; CRP: C-reactive protein; CHOL: total cholesterol; LDL-C: low-density lipoprotein cholesterol; HDL-C: high-density lipoprotein cholesterol; TG: triglycerides; GLP-1: glucagon-like peptide 1; PYY: peptide YY; HOMA-IR: homeostasis model assessment for insulin resistance; CO: carbon monoxide. * *p* values with paired samples *t*-test. ** *p* values with Wilcoxon matched-pair signed-rank test. Data are presented as n (%), mean ± standard deviation, or median (interquartile range).

**Table 3:** Comparative analysis of physical activity levels (MET-min/week) among participants.

Variables	Baseline (0)	2.5 Months	4 Months	*p*-Values 0 vs. 2.5	*p*-Values 0 vs. 4	*p*-Values 2.5 vs. 4
MET-min/week	537 (264; 1930)	701 (346; 2633)	1182 (485; 1903)	0.041 *	0.016 *	0.653 *

MET: metabolic equivalent of task. * *p* values with Wilcoxon matched-pair signed-rank test. Data are presented as median (interquartile range).

**Table 4:** Correlations between changes in peptide levels and weight-related parameters during the study.

Variable Changes	Leptin	GLP-1	Ghrelin	Adiponectin	Resistin	PYY	HOMA-IR
Weight	0.44 *	−0.10	−0.40 *	−0.29	0.28	0.10	0.15
BMI	0.47 **	−0.11	−0.38 *	−0.29	0.27	0.09	0.10
WC	0.45 *	−0.29	−0.29	−0.07	0.22	0.30	−0.02
RMR	0.27	0.10	−0.02	−0.20	−0.07	−0.16	−0.16
CHOL	0.08	−0.35 *	−0.54 **	−0.12	−0.06	−0.43 *	0.11
LDL-C	0.02	−0.41 *	−0.39 *	−0.17	−0.04	−0.37 *	0.08
HDL-C	−0.08	0.19	−0.07	0.49 **	−0.05	−0.24	0.11
TG	−0.13	0.07	−0.49 **	−0.29	0.13	−0.18	0.17
MET-min/week	−0.33	−0.18	0.34	−0.26	−0.15	0.09	0.13

GLP-1: glucagon-like peptide 1; PYY: peptide YY; HOMA-IR: homeostasis model assessment for insulin resistance; BMI: body mass index; WC: waist circumference; RMR: resting metabolic rate; CHOL: total cholesterol; LDL-C: low-density lipoprotein cholesterol; HDL-C: high-density lipoprotein cholesterol; TG: triglycerides; MET: Metabolic Equivalent of Task. Table values represent “rho” (ρ), which is Spearman’s correlation coefficient. * *p* < 0.05. ** *p* < 0.01.

## Data Availability

The data presented in this study are available on request from the corresponding author.
